# Single-Cell Transcriptomics Reveals Killing Mechanisms of Antitumor Cytotoxic CD4^+^ TCR-T Cells

**DOI:** 10.3389/fimmu.2022.939940

**Published:** 2022-07-19

**Authors:** Yanling Liang, Qumiao Xu, Songming Liu, Jie Li, Fei Wang, Ziyi Li, Lijuan Liao, Yuting Lu, Yijian Li, Feng Mu, Hai-Xi Sun, Linnan Zhu

**Affiliations:** ^1^ College of Life Sciences, University of Chinese Academy of Sciences, Beijing, China; ^2^ Beijing Genomics Institute (BGI)-Shenzhen, Shenzhen, China; ^3^ Beijing Genomics Institute (BGI), Shenzhen, China; ^4^ Beijing Genomics Institute (BGI)-Beijing, Beijing, China; ^5^ Biomedical Pioneering Innovation Center (BIOPIC), Peking University, Beijing, China; ^6^ Institute of Cancer Research, Shenzhen Bay Laboratory, Shenzhen, China

**Keywords:** cytotoxic CD4^+^ T, TCR-T, single-cell RNA sequencing, adoptive T cell therapy, LTA

## Abstract

T cell receptor-engineered T cells (TCR-Ts) have emerged as potent cancer immunotherapies. While most research focused on classical cytotoxic CD8^+^ T cells, the application of CD4^+^ T cells in adoptive T cell therapy has gained much interest recently. However, the cytotoxic mechanisms of CD4^+^ TCR-Ts have not been fully revealed. In this study, we obtained an MHC class I-restricted MART-1_27-35_-specific TCR sequence based on the single-cell V(D)J sequencing technology, and constructed MART-1_27-35_-specific CD4^+^ TCR-Ts and CD8^+^ TCR-Ts. The antitumor effects of CD4^+^ TCR-Ts were comparable to those of CD8^+^ TCR-Ts *in vitro* and *in vivo*. To delineate the killing mechanisms of cytotoxic CD4^+^ TCR-Ts, we performed single-cell RNA sequencing and found that classical granule-dependent and independent cytolytic pathways were commonly used in CD4^+^ and CD8^+^ TCR-Ts, while high expression of *LTA* and various costimulatory receptors were unique features for cytotoxic CD4^+^ TCR-Ts. Further signaling pathway analysis revealed that transcription factors Runx3 and Blimp1/Tbx21 were crucial for the development and killing function of cytotoxic CD4^+^ T cells. Taken together, we report the antitumor effects and multifaceted killing mechanisms of CD4^+^ TCR-Ts, and also indicate that MHC class I-restricted CD4^+^ TCR-Ts could serve as potential adoptive T cell therapies.

## Introduction

T cell receptors (TCRs) are responsible for recognizing antigens in the form of processed peptides presented by major histocompatibility complex (MHC), providing the first signal to trigger T cell signaling transduction. T cells modified with tumor-reactive TCRs are endowed with specificity to tumor antigens, therefore considered powerful weapons to fight against tumors. In the past decades, adoptive cellular therapies using TCR-engineered T cells (TCR-Ts) mediated durable tumor regression in patients with melanoma, synovial sarcoma and myeloma (1, 2), and a growing number of clinical trials in various solid cancers are currently underway (3–5).

Tumor associated antigens (TAAs), which are overexpressed on neoplastic cells but have low expression level on normal cells, represent a major class of tumor antigens. In melanoma, one of the first TAAs verified was melanocyte lineage-specific protein (MART-1), and its immunodominant epitope MART-1_27-35_ was recognized by HLA-A2-restricted cytotoxic T lymphocytes (CTLs) derived from some melanoma patients (6). Although CD8^+^ CTLs are best known as responders to eradicate malignant cells, studies have shown that CD4^+^ cytotoxic T cells existing naturally in the context of infectious diseases and cancers could directly kill infected or cancer cells (7–10). For adoptive cell therapy, autologous neoantigen-reactive CD4^+^ T cells mediated effective disease regression in patients with metastatic cholangiocarcinoma (9, 11). In a clinical study for metastatic cancers, four objective responses were observed among 17 patients treated by CD4^+^ TCR-Ts targeting an MHC class II-restricted epitope (12).

One of the challenges for CD4+ TCR-T therapy resulted from the rare expression of MHC class II molecules on most tumor cells (13, 14). An in-depth analysis of CD4^+^ T cells infiltrating human melanoma specimens revealed the diverse targets of anti-tumor CD4^+^ TCRs, including MHC class II-restricted neoantigens and HLA class I-restricted TAAs (15). Besides, studies showed that MHC class I-restricted tumor-specific TCR could be harnessed to program CD4^+^ T cells with antitumor effector functions (16–18). MHC class I-restricted CD4^+^ TCR-Ts were found to synthesize Th1 cytokines and exhibit cytolytic effector functions in a human melanoma model (19). Infusion of cell products composed of CD4^+^ and CD8^+^ TCR-Ts targeting an MHC class I-restricted HPV-16 E7 epitpope led to objective clinical responses in patients with metastatic HPV-16^+^ cancers. The phenotype, efficacy and persistence of infusion products were characterized *in vitro* and after engraftment, while the role of CD4^+^ TCR-Ts was not specifically defined or compared with CD8^+^ TCR-Ts (5). Despite previous work provided insights on the cytolytic features of CD4^+^ T cells (20), the effector mechanisms of MHC class I-restricted CD4^+^ TCR-Ts have not been fully revealed. How cytotoxic CD4^+^ T cells differ from conventional CD8^+^ T cells requires further study.

In this study, we constructed TCR-Ts using an MHC class I-restricted TCR targeting MART-1_27-35_ identified in house, and demonstrated that both CD4^+^ TCR-Ts and CD8^+^ TCR-Ts had effective antitumor cytotoxic activities *in vitro* and *in vivo*. We further deciphered the shared and unique cytotoxic mechanisms of CD4^+^ and CD8^+^ TCR-Ts after antigen stimulation through single-cell RNA sequencing (scRNA-seq) analysis and experimental validation. Our results suggested that classical granule-dependent and independent cytotoxic pathways were commonly used in CD4^+^ and CD8^+^ TCR-Ts, while high expression of lymphotoxin-α (LT-α) and various costimulatory receptors were unique features for cytotoxic CD4^+^ TCR-Ts. Furthermore, single-cell transcriptomic profiles of CD4^+^ TCR-Ts from different durations of antigen stimulation showed a clear differentiation trend towards cell clusters with strong cytotoxic functions. Together, using a tumor-specific MHC class I-restricted TCR, our study depicted the functional and molecular characteristics of cytotoxic CD4^+^ TCR-Ts at the single-cell transcriptomic level, which revealed the multifaceted cytotoxic pathways for CD4^+^ TCR-Ts.

## Results

### MHC Class I-Restricted CD4^+^ TCR-Ts Exerted Antitumor Cytotoxic Functions *In Vitro* and *In Vivo*

**Figure 1:**
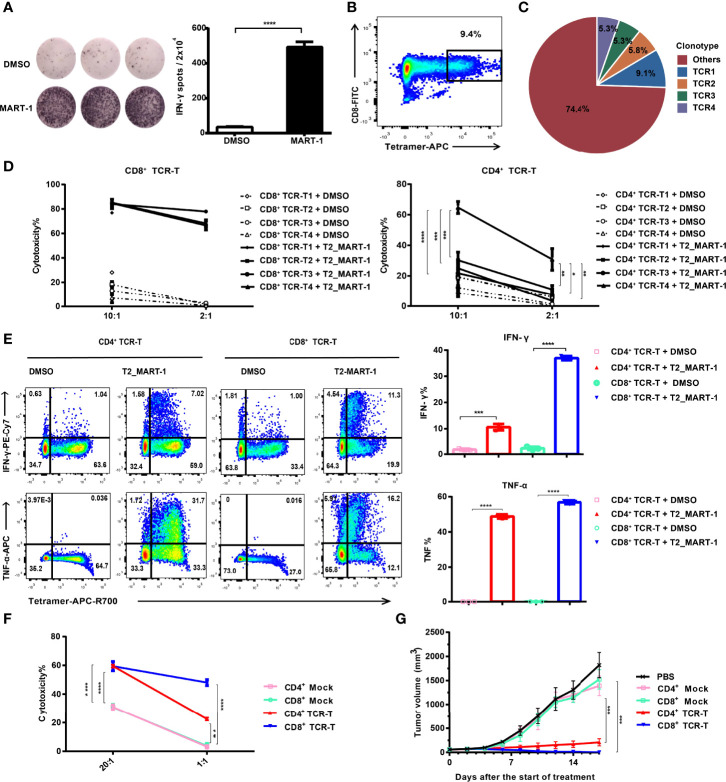
Construction and functional validation of MART-1_27-35_-specific CD4^+^ TCR-Ts. **(A)** IFN-γ ELISPOT results after co-culture of CD8^+^ T cells with MART-1_27-35_ or DMSO-pulsed T2 cells. **(B)** FACS sorting of MART-1_27-35_-specific T cells stained with tetramers. **(C)** Pie chart of TCR clonotype frequency from single-cell V(D)J sequencing. **(D)** Cytotoxicity of CD8^+^ TCR-Ts and CD4^+^ TCR-Ts expressing top4 TCR clonotypes to MART-1_27-35_ or DMSO-pulsed T2 cells. **(E)** Cytokine production by CD8^+^ TCR-Ts and CD4^+^ TCR-Ts stimulated with MART-1_27-35_ or DMSO-pulsed T2 cells. **(F)** Killing of A375_MART-1_ melanoma cell line by CD4^+^ and CD8^+^ TCR-Ts. **(G)** Mean tumor growth curves of five groups after treatment. NOG mice were implanted with A375_MART-1_ subcutaneously. Mice were divided into five groups after 7 days, and received different treatment through peritumoral injection at day 0 (start of treatment) and day 7: PBS (n = 5), CD4^+^ mock T (n = 3, 3.0×10^7^), CD4^+^ TCR-T (n=5, 3.0×10^7^), CD8^+^ mock T (n = 5, 3.0×10^7^) and CD8^+^ TCR-T (n = 5, 3.0×10^7^). Tumor volume was calculated every 2 days. **(A-F)**, groups were compared with a two-sided, unpaired t-test. **(G)**, groups were compared with the one-way ANOVA analysis. Error bars denote the SD. *:P < 0.05; **:P < 0.01; ***:P < 0.001; ****:P < 0.0001.

To obtain MHC class I-directed tumor-specific TCR, we induced MART-1_27-35_-specific CTLs from an HLA-A:0201 healthy donor. IFN-γ ELISPOT assay showed that MART-1_27-35_-pulsed T2 cells induced significant IFN-γ production from CTLs (**Figure 1A**). To obtain the TCR sequences of MART-1_27-35_-specific CTLs, we sorted MART-1_27-35_/MHC tetramer-positive CTLs (**Figure 1B**), followed by single-cell V(D)J sequencing. Full-length, paired V(D)J sequences were assembled from a total of 790 cells, which consisted of 245 TCR clonotypes (**Figure 1C**). The top4 TCR clonotypes with frequencies over 5%, namely, TCR1, TCR2, TCR3 and TCR4 (**Table S1**) were selected to construct TCR-Ts for functional investigation.

HLA-A:0201 healthy donor-derived CD4^+^ and CD8^+^ T cells were transduced with lentiviral vectors carrying TCR expression cassettes (**Figure S1A**) respectively. After expansion, cells were labeled by anti-mouse TCRβ antibodies and sorted by flow cytometry to achieve equal expression of exogenous TCRs (~100% positive for exogenous TCR, **Figure S1B**). Functions of CD4^+^ and CD8^+^ TCR-Ts were evaluated *in vitro* through incubation with antigen-loaded target cells. Both CD4^+^ TCR-Ts and CD8^+^ TCR-Ts generated from these four TCR clonotypes (TCR-T_1_, TCR-T_2_, TCR-T_3_, TCR-T_4_) displayed significant cytotoxicity to MART-1_27-35_-pulsed T2 cells at 2:1 and 10:1 effector-to-target ratios compared with the control group (T2 cells loaded with DMSO, **Figure 1D**). The cytotoxic activities of CD8^+^ TCR-Ts were similar for the four TCR clonotypes. By contrast, CD4^+^ TCR-Ts from TCR4 presented significantly stronger killing towards target cells, compared with the other three kinds of CD4^+^ TCR-Ts (**Figure 1D**), suggesting that TCR4 might have a higher affinity to the MART-1_27-35_/MHC complex. Based on the results, we speculated that TCR4 was likely CD8 coreceptor-independent, while the other three TCRs were dependent on the CD8 coreceptor for effective activation. Signature cytokines for cytotoxic T cells or Th1 cells, IFN-γ and TNF-α were substantially upregulated in CD4^+^ TCR-Ts and CD8^+^ TCR-Ts upon stimulation by MART-1_27-35_-pulsed T2 cells (**Figure 1E**).

CD4^+^ TCR-T_4_ and CD8^+^ TCR-T_4_ were then used for following functional validation and phenotypic characterization. Specific killing activities from both CD4^+^ and CD8^+^ TCR-Ts to the MART-1_27-35_-overexpressing A375 cells were detected by LDH cytotoxicity assay and real-time cell analysis (**Figures 1F**, **1SC**), while CD8^+^ TCR-Ts initiated faster killing of target cells (**Figure 1SC**). To evaluate *in vivo* antitumor efficacy of CD4^+^ TCR-Ts, we established a xenograft NOG (NOD/Shi-*scid*/IL-2Rγ^null^) mouse model with MART-1_27-35_-overexpressing A375 cells. CD4^+^ or CD8^+^ TCR-Ts were transferred to mice through peritumoral injection on day 7 and day 14 after tumor implantation. Compared with mock T cells (CD4^+^ or CD8^+^ T cells transduced with lentivirus carrying *GFP* gene), CD4^+^ or CD8^+^ TCR-Ts mediated effective tumor eradication *in vivo* (**Figures 1G**, **1SD**). Together, we have generated MHC class I-restricted tumor-specific CD4^+^ TCR-Ts that were capable of antitumor effector functions comparable to CD8^+^ TCR-Ts, such as secretion of inflammatory cytokines and direct lysis of target cells.

### “TCR-Activated” Cytotoxic Clusters Were Identified in CD4^+^ and CD8^+^ TCR-Ts

**Figure 2:**
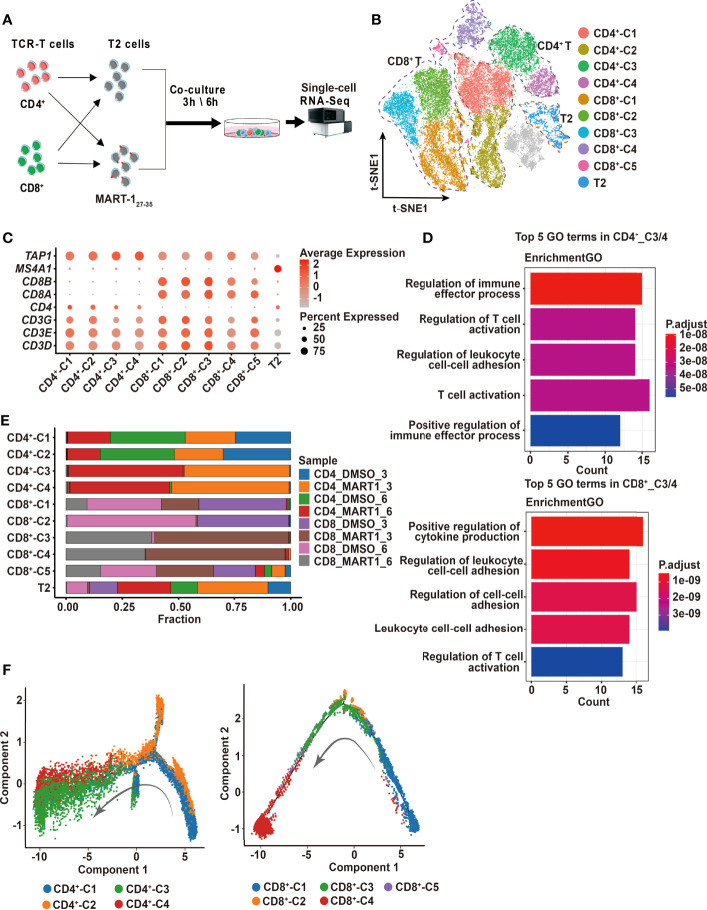
Single-cell profiling of CD4^+^ and CD8^+^ TCR-Ts cocultured with target cells. **(A)** Flow chart of the single-cell RNA sequencing experimental design. **(B)** t-SNE visualization of the expression profiles of the 43,368 cells that passed quality control. Clusters pertaining to CD8^+^ T cells, CD4^+^ T cells and T2 cells were illustrated by dotted lines. Grey refers to undefined cells. **(C)** Dot plot depicting the average expression and percent of cells expression maker genes for each given cluster. *CD3D/E/G* for T cells, *CD4* for CD4^+^ T cells, and *CD8A/B* for CD8^+^ T cells. *TAP1* and *MS4A1* are negative and positive markers of T2 cells, respectively. **(D)** GO pathway analysis of CD4^+^_C3/C4, CD8^+^_C3/C4 compared with CD4^+^_C1/C2 or CD8^+^_C1/C2/C5 (FC > 1.0, P < 0.05). The color key from red to blue indicates P values from low to high. Count represents the number of genes enriched to this GO entry from the input gene for enrichment analysis. **(E)** Stacked bar chart of sample proportions in each cluster. **(F)** Pseudotime trajectory plots of CD4^+^ T (left) or CD8^+^ T cells (right) across the pseudotime trajectory. Each point corresponds to one single cell. Each color represents one cell cluster. GO, gene ontology. t-SNE, t-distributed stochastic neighbor embedding.

Next, we sought to investigate the cytotoxic mechanisms of CD4^+^ and CD8^+^ TCR-Ts at the single-cell transcriptomic level. CD4^+^ and CD8^+^ TCR-Ts were incubated with MART-1_27-35_-pulsed T2 cells or DMSO-pulsed T2 cells at effector-to-target ratio of 10:1, for 3 or 6 hours respectively. Single-cell RNA sequencing was performed on 8 samples (**Figure 2A**, **Table S2**), and a total of 54,166 cells were obtained after single-cell data processing, resulting in a total of 43,368 high-quality cells after filtering (**Figure 2B**). Eleven cell clusters were identified through unsupervised clustering, which were assigned to CD4^+^_C1/C2/C3/C4, CD8^+^_C1/C2/C3/C4/C5, and T2 cells according to the expression of marker genes, including *CD3D/E/G* for T cells*, CD4* for CD4^+^ T cells, *CD8A/B* for CD8^+^ T cells, *MS4A1* and lack of *TAP1* expression for T2 cells (**Figure 2C**).

To determine the activation states and functions of cell clusters, we analyzed the top10 differentially expressed genes (DEGs) from each cluster, showing distinct transcriptomic profiles among CD4^+^ and CD8^+^ clusters (**Figure S2A**, top5 DEGs were displayed). Genes related to effector functions or activated states, such as *CD40LG*, *LTA*, *IFNG* were upregulated in CD4^+^_C3/C4 or CD8^+^_C3/C4, indicating that these clusters were responding to TCR activation (**Figure S2A**). We then performed the differential expression analysis comparing CD4^+^_C3/C4 or CD8^+^_C3/C4 with their remaining clusters. Genes upregulated in CD4^+^_C3/C4 or CD8^+^_C3/C4 included pro-inflammatory cytokines and chemokines, and were significantly enriched in T cell activation and effector functional pathways (**Figures 2D**, **S2B**). Besides, cell compositions in each cluster demonstrated that cells of CD4^+^_C3/C4 or CD8^+^_C3/C4 were predominantly from CD4^+^ or CD8^+^ TCR-Ts stimulated with MART-1_27-35_-pulsed T2 cells (CD4_MART1_3, CD4_MART1_6, CD8_MART1_3, CD8_MART1_6), while other clusters contained cells from various sample groups (**Figure 2E**), which also suggested CD4^+^_C3/C4 and CD8^+^_C3/C4 responsive to antigen stimulation.

To understand the cell state transitions of CD4^+^ or CD8^+^ T cells, we used an unsupervised inference method Monocle2 to perform pseudo-chronological analysis. CD4^+^_C3/C4 or CD8^+^_C3/C4 were mostly located at the middle to late differential stages (**Figure 2F**), which was in line with their activated states. Analysis of representative gene sets for activation/exhaustion, co-stimulation or cytotoxicity showed that CD4^+^_C3/C4 and CD8^+^_C3/C4 were more active in these effector functions than other clusters, and revealed different levels of co-stimulatory or cytotoxic activities between CD4^+^_C3/C4 and CD8^+^_C3/C4 (**Figures S2C-E**). Therefore, CD4^+^_C3/C4 and CD8^+^_C3/C4 were defined as “TCR-activated” clusters, which differentiated into effector cells with cytotoxic functions, while CD4^+^_C1/C2 and CD8^+^_ C1/C2/C5 mostly contained non-activated T cells, including cells stimulated with DMSO-pulsed T2, nontransduced T cells and TCR-Ts at the resting state.

### Single-Cell Transcriptomic Analysis Revealed Common and Different Cytotoxic Mechanisms of CD4^+^ and CD8^+^ TCR-Ts

In order to discover the shared and distinct cytotoxic mechanisms of CD4^+^ and CD8^+^ TCR-T cells, we graphed the 220 and 127 significantly up-regulated genes from CD4^+^_C3/C4 and CD8^+^_C3/C4 compared to non-activated T cell clusters, among which 95 up-regulated genes were shared in “TCR-activated” clusters, which included well-known effector genes, e.g., *FASLG*, *TNF*, *IFNG*, and *GZMB*, *etc.* (**Figure S3A**). Thus, both CD4^+^ and CD8^+^ TCR-T cells exerted cytotoxic functions through the release of cytotoxic proteins as well as granule-independent mechanisms. Gene set enrichment analysis (GSEA) of apoptosis pathways in T2 target cells showed that signature genes related to three types of apoptosis pathways were enriched in MART-1_27-35_-pulsed T2 cells incubated with CD4^+^ or CD8^+^ TCR-T cells (**Figures S3C, D**), and CD4^+^ TCR-T cells tended to induce the lysis of target cells *via* hallmark apoptosis and necrotic cell death pathways (**Figure S3B**).

**Figure 3:**
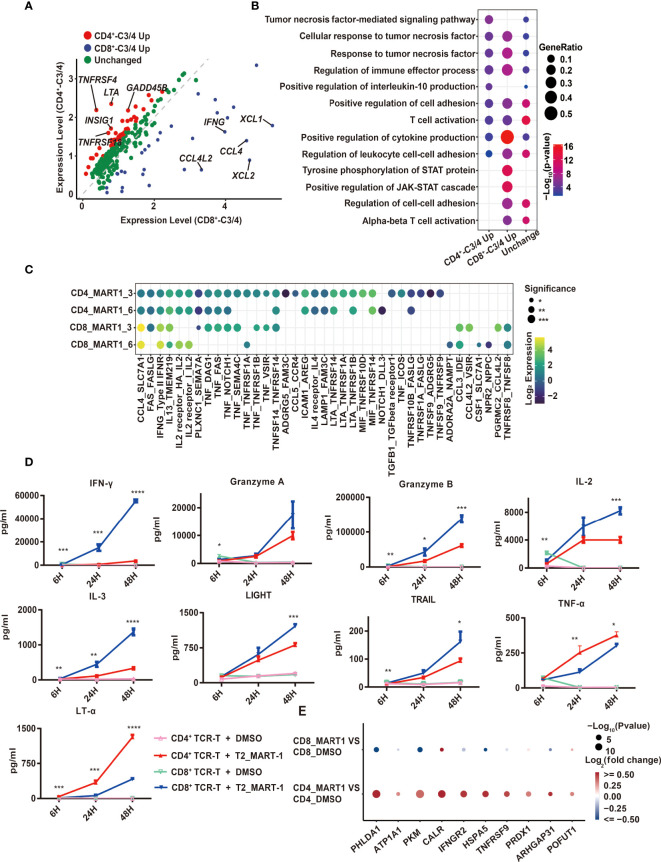
Comparison of CD4^+^ and CD8^+^ TCR-T cytotoxic programs through scRNA-seq analysis and cytokine release measurement. **(A)** Volcano map of differentially expressed genes between CD4^+^_C3/C4 and CD8^+^ _C3/C4 using 252 genes. Red dots indicate genes upregulated in CD4^+^_C3/C4 and blue dots indicate genes upregulated in CD8^+^_C3/C4. Green dots indicate genes with no significant change between CD4^+^_C3/C4 and CD8^+^_C3/C4 (FC>1.5, P < 0.05). **(B)** Representative GO terms enriched based on differentially expressed genes as shown in **(A)**. The circle size indicates the gene ratio of the gene number enriched in the GO term divided by the total genes in the group (CD4_C3/C4 Up, CD8_C3/C4 Up, Unchanged). The color key from red to blue indicates P values (P < 0.05) from low to high. **(C)** Cell-cell communication analysis of TCR-Ts with T2 target cells (the ligand expressed in T cell functional clusters while the receptor expressed in target cells). The circle color indicates the log-scaled (base = 2) expression of each ligand-receptor pair, the circle size indicates the significance. **(D)** Cytokine secretion of TCR-Ts stimulated with MART-1_27-35_ or DMSO-pulsed T2 cells measured by Luminex. Statistical significance between the CD4^+^ and CD8^+^ TCR-Ts stimulated with MART-1_27-35_ groups was determined by an unpaired, two-tailed Student’s t-test. *: P < 0.05, **: P < 0.01, ***: P < 0.001. GO, gene ontology. **(E)** Genes downstream of LTA signaling pathway were significantly up-regulated in T2 cells.

Using the combined set of 252 up-regulated genes, we compared their gene expression directly between CD4^+^_C3/C4 and CD8^+^_C3/C4, which identified genes with higher expression in CD4^+^_C3/C4 (CD4^+^_C3/C4 Up, FC>1.5, P<0.05) or CD8^+^_C3/C4 (CD8^+^_C3/C4 Up, FC>1.5, P<0.05), as well as genes with similar expression levels (Unchanged) (**Figure 3A**). The pathway analysis showed that three groups of genes were similarly enriched in T cell activation, cytokine production, tumor necrosis factor-related pathways, and regulation of leukocyte adhesion (**Figure 3B**). CD8^+^_C3/C4 Up involved inflammatory chemokines, cytokines, and cytotoxic proteins, as well as pathways related to STAT protein tyrosine phosphorylation and positive regulation of the JAK-STAT cascade. This is consistent with the higher cytotoxic activities for CD8^+^ TCR-Ts (**Figures 1D, E**). CD4^+^_C3/C4 Up included costimulatory receptors and cytokines in the tumor necrosis factor (TNF) family. *TNFRSF4* (OX40) and *TNFRSF18* (GITR) were specifically highly expressed in CD4^+^_C3/C4 (**Figure 3A**), which are involved in the activation, proliferation and differentiation of T cells *in vivo*, and are also reported to play important roles in maintaining and promoting the function of effector T cells (21, 22). The co-stimulation signals might be beneficial for the long-term and sustained antitumor immune response of CD4^+^ TCR-Ts.

The expression of TNF family cytokines was high in CD4^+^_C3/C4, especially *LTA* (**Figure 3A**), which encodes lymphotoxin-α (LT-α) regulating cell survival, proliferation and apoptosis (23). Consistent with this, the transcription regulons *IRF4* and *IRF8*, which were reported to regulate *LTA* transcription (24, 25), also had higher activities in CD4^+^_C3/C4 (**Figure S3E**). In addition, when examining cellular interactions between T cell clusters and T2 cells, we found strong interactions of *LTA* from CD4^+^ TCR-Ts with corresponding receptors *TNFRSF14*, *TNFRSF1A* and *TNFRSF1B* on T2 cells (**Figure 3C**). Similarly, interactions between *TNF* with its receptors *TNFRSF1A* and *TNFRSF1B* were enriched for CD4^+^ TCR-Ts and T2 cells. As the importance of TNF-α in direct antitumor activity with CD4^+^ T cells was recently verified (26), our results also uncovered *LTA* and *TNF* as key effector molecules for cytotoxic CD4^+^ TCR-Ts, which were previously unrecognized mechanisms in CD4^+^ T cells.

Production of various effector cytokines from CD4^+^ and CD8^+^ TCR-Ts stimulated by MART-1_27-35_-pulsed T2 cells was validated using Luminex assay. Multiple inflammatory cytokines, such as IFN-γ, IL-3, IL23, were secreted more from CD8^+^ TCR-Ts (**Figure 3D**, **S3F**). Granzyme A and granzyme B were produced higher in CD8^+^ TCR-Ts, while CD4^+^ TCR-Ts also released a substantial amount of these cytotoxic proteins (**Figure 3D**). TNF superfamily proteins, such as LIGHT and TRAIL were produced at higher levels from CD8^+^ TCR-Ts at 48h, but TNF-α and LT-α were secreted more from CD4^+^ TCR-Ts (**Figure 3D**), in accordance with single-cell transcriptomic analysis. These data confirmed the functional convergences and differences for cytotoxic CD4^+^ and CD8^+^ TCR-Ts.

In addition to elevated LTA expression at both mRNA level and protein level in CD4+ TCR-Ts, many genes downstream of LTA signaling pathway were significantly up-regulated in T2 cells of CD4-MART-1 group, including *CALR, HSPA5, PRDX1, IFNGR2, TNFRSF9, GNA15, PHLDA1, SERPINB2 and ARHGAP31* (**Figure 3E**). PHLDA1 has been reported to induce apoptosis in various cells including T cells, endothelial cells and melanoma cells (27, 28). ATP1A1 may induce renal cell carcinoma cell apoptosis by mediating the Raf/MEK/ERK signaling pathway (29). Calreticulin, the calcium-binding chaperone encoded by *CALR*, participated in pre-apoptotic and early stages of apoptotic cells and modulated immune responses to dying cells (30, 31).

Together, we depicted the cytotoxic characteristics of CD4^+^ and CD8^+^ TCR-Ts from DEG analysis, cellular communication and transcriptional regulatory networks based on scRNA analysis. Overall, CD4^+^ TCR-Ts shared similar cytotoxic mechanisms with CD8^+^ TCR-Ts, with unique features of higher costimulatory signals and LT-α pathway activities.

### CD4^+^ TCR-Ts Displayed Mixed Th1/Th2 Phenotypes and Cytotoxic Signatures

**Figure 4:**
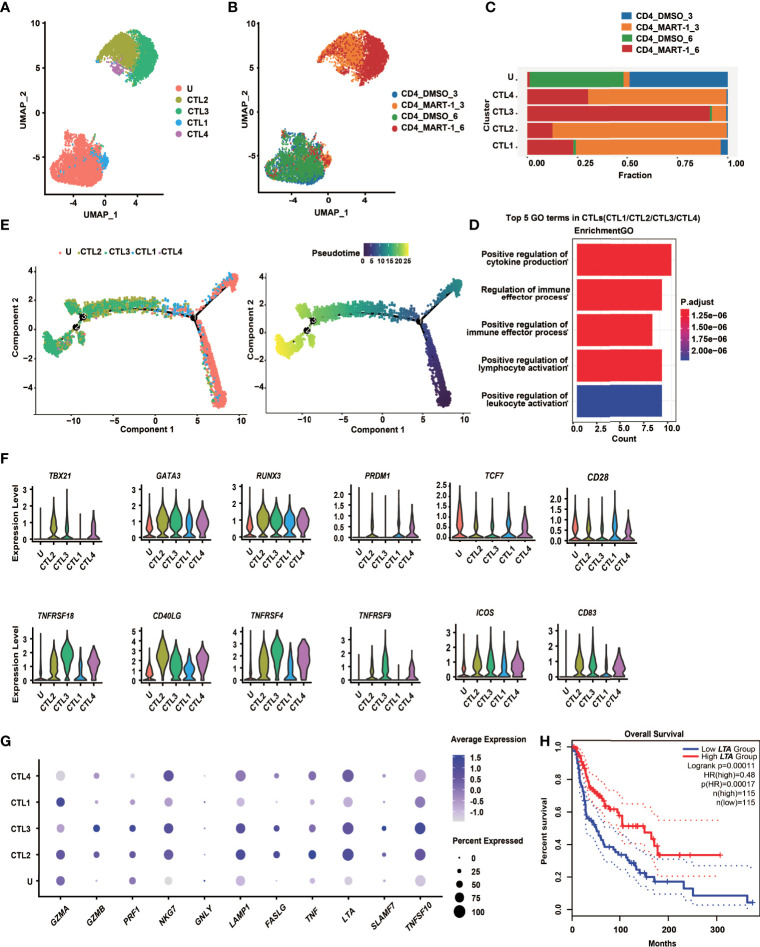
CD4^+^ TCR-T clusters showed temporal transcriptome profiles, mixed Th1/Th2 phenotypes and unique cytotoxic signatures. **(A)**UMAP projection of the expression profiles of the 7825 CD4^+^ TCR-T cells. **(B)** UMAP manifolds colored by sample origins. **(C)** Stacked bar chart of sample origins in each CD4^+^ TCR-T cluster. **(D)** GO pathway analysis of CTL1/2/3/4 compared with cluster U. Top 30 DEGs were used for analysis. Top 5 GO-BP terms were displayed. The color key from red to blue indicates P values (P < 0.05) from low to high. Count: The number of genes enriched to this GO entry from the input gene for enrichment analysis. **(E)** Pseudotime trajectory plot of CD4^+^ TCR-T cells across the pseudotime trajectory. **(F)** Violin plots show expression of selected transcription factors and costimulatory molecules in CD4^+^ TCR-T clusters. **(G)** Dot plots show expression of representative cytotoxic genes in CD4^+^ TCR-T clusters. **(H)** Kaplan-Meier overall survival curves of TCGA SKCM patients grouped by LTA expression. P value was calculated by a log-rank test. DEG, differential expressed gene; GO, gene ontology; UMAP, uniform approximation and projection; BP, biological process.

To explore the cell state heterogeneity within cytotoxic CD4^+^ TCR-Ts, we clustered single-cell transcriptomics of CD4^+^ TCR-Ts separately. Five clusters were resolved, and based on the sample origin, the nonactivated cluster dominated by CD4^+^ TCR-Ts stimulated with DMSO-treated T2 cells (Cluster U), as well as four CTL clusters (CTL1/2/3/4) composed mostly of CD4^+^ TCR-Ts stimulated with MART-1_27-35_-pulsed T2 cells were defined (**Figures 4A-C**). Time-dependent transcriptomic profiles were observed in CTL clusters from CD4^+^ TCR-Ts stimulated with antigen for 3 or 6 hours (**Figure 4B**). To infer the biological processes associated with CTL clusters, we isolated the top 30 up-regulated genes for each cluster and performed Gene Ontology (GO) enrichment analysis. Compared with nonactivated cluster U, CTL1/2/3/4 were enriched with functional pathways like cell activation, cytokine production, cell killing, and positive immune regulation (**Figures 4D**, **S4A-D**). Pseudo-chronological analysis suggested a differentiation path of nonactivated cluster U to CTL1, CTL2/4, and CTL3, with CTL3 primarily at the latest stage (**Figure 4E**). These results showed that CD4^+^ TCR-Ts differentiated into effector T cells driven by antigen stimulation, and CTL3 (mainly consisting of the sample stimulated with antigen for 6 hours) was at the latest differentiation stage with the most significant activation of various functional pathways (**Figure S4C**).

Transcription factors essential for CD4^+^ T cell lineage development were expressed at different levels in CD4^+^ TCR-T clusters (**Figure 4F**). Both *GATA3* and *TBX21* were highly expressed in CTL2/3/4, indicating that CD4^+^ TCR-Ts displayed Th1/Th2 mixed phenotypes, consistent with the *in vitro* assay showing that multiple Th1 cytokines, such as IL-2, IFN-γ and LT-α, as well as Th2 cytokines like IL-4, IL-5, IL-13 were produced from CD4^+^ TCR-Ts (**Figures 3D**, **S3F**). *RUNX3*, a key transcription factor for cytotoxic CD4^+^ T cells (32, 33), was upregulated in CTL1/2/3/4 and might promote the differentiation of CD4^+^ T cells towards the cytotoxic lineage. Additionally, the necessity of *PRDM1* in the differentiation of cytotoxic CD4^+^ T cells has been reported in mice and human (34, 35).

The costimulatory molecules, such as *TNFRSF4* (OX40), *TNFRSF9* (4-1BB) and *TNFRSF18* (GITR) were highly expressed in CTL2/3/4 (**Figure 4F**), which provided the second signals for T cell activation, and contributed to the persistence of CD4^+^ T cells (21, 22). Upregulation of *FASLG* and genes encoding cytolytic granule-associated molecules (*GZMA*, *GZMB*, *PRF1*, *LAMP1* and *NKG7*) in CTL2/3/4 (**Figure 4G**) represented two types of classical cytotoxic mechanisms, inducing the apoptosis of target cells *via* FAS/FASLG signaling pathways and granule-dependent pathways.

Besides, we also noted genes upregulated in cytotoxic CD4^+^ TCR-Ts that might be of value as phenotypic markers. For example, granule-associated molecule natural killer cell granule protein-7 (*NKG7*) was recently discovered by Wen et al. as a crucial intrinsic factor for CD8^+^ T cells to accomplish efficient antitumor cytotoxicity (36). Our results suggested that it could also be critical for cytotoxic CD4^+^ TCR-Ts, given that expression of *NKG7* was highly upregulated in CTL2/3/4 (**Figure 4G**). In addition, a newly identified target *SLAMF7* (signaling lymphocytic activation molecule F7) which was associated with cytotoxic activity of MHC class II-restricted CD4^+^ T cells (37), was expressed in CTL2/3/4, especially in CTL3 (**Figure 4G**), indicating that induction of *SLAMF7* was correlated with cytotoxic function, and it might be a unique signature for cytotoxic CD4^+^ TCR-Ts.

Moreover, effector molecules from the TNF superfamily, such as TNF-α, LT-α and TRAIL, which were known to convey cytotoxic functions might serve as additional cytotoxic programs utilized by CD4^+^ T cells. The expression levels of *TNF*, *LTA*, and *TRAIL* were high in CTL2/3/4 (**Figure 4G**), and substantial levels of cytokines released from CD4^+^ TCR-Ts were also detected by *in vitro* functional assays (**Figures 3D**, **S3F**). Notably, expression of *LTA* was positively correlated with favorable survival in melanoma patients using a SKCM (skin cutaneous melanoma) dataset from TCGA database (**Figure 4H**, HR(high)=0.48, p(HR)=0.00017), suggesting the important role of *LTA* in antitumor immune responses. A gene set of essential effector molecules were presented as the cytotoxic signatures for CD4^+^ TCR-Ts (**Figure 4G**).

In conclusion, CD4^+^ TCR-Ts presented temporal transcriptome profiles after antigen stimulation, and differentiated along the mixed Th1/Th2 paths with the acquisition of cytotoxic functions. The results also showed that multifaceted cytotoxic mechanisms were utilized by CD4^+^ TCR-Ts to induce cell death of target cells, providing a comprehensive molecular map as well as unique cytotoxic features of MHC class I-restricted CD4^+^ TCR-Ts.

## Discussion

CD4^+^ T cells are highly versatile multifunctional cells that together with CD8^+^ cytotoxic T cells constitute the T cell effector immune system. CD4^+^ T cells can differentiate into one of multiple functional subtypes in response to relevant signals, which in turn enables them to act as the main central coordinator among appropriate effector immune cells (38). Historically, it was well acknowledged that cytotoxic CD8^+^ T cells take the front seat in mediating direct anti-tumor immune responses, while CD4^+^ T cells play important roles in promoting the activation, proliferation, differentiation and maintenance of CD8^+^ T cell pools (39–41). Over the past decades, accumulating research has noted the cytotoxic functions of CD4^+^ T cells in cancers (8–10, 37), which displayed heterogeneous phenotypes with functional capacity of direct tumor lysis through MHC class II-dependent pathways.

Since MHC class II-restricted antigens naturally recognized by CD4^+^ T cells were poorly presented on most tumor cells (42), CD4^+^ T cells engineered with an MHC class I-restricted TCR have been evaluated as an attractive strategy to exert antitumor effector functions. Previous work suggested that MHC class I-restricted TCRs with high functional avidity were CD8 coreceptor independent, which directed CD4^+^ T cells to produce typical Th1 cytokines and cytotoxic proteins for tumor elimination (17, 19, 20). For the four MART-1_27-35_-specific TCRs identified in our study, we showed that TCR4 was likely CD8 coreceptor independent, as it prompted significant effector functions, including cytokine release and cytotoxicity when transferred to CD4^+^ T cells (**Figures 1D-G**).

By far, the cytolytic activity of CD4^+^ T cells was mostly depicted *via* traditional functional assays which were limited by the number of analytes or pathways. A recent study performed scRNA sequencing on CD4^+^ T cells overexpressing a CD8 coreceptor dependent MHC class I-restricted TCR and the CD8αβ coreceptor, and identified differential pathway usage and T cell differentiation status through unbiased single-cell transcriptional analysis (43). However, the transcriptomic profile of CD4^+^ T cells with a CD8 coreceptor independent TCR remains unclear. In this study, we applied parallel scRNA sequencing on CD4^+^ and CD8^+^ TCR-Ts overexpressing a CD8 coreceptor independent TCR (TCR4). By comparing antigen stimulation with control, we identified CD4^+^ or CD8^+^ “TCR-activated” clusters, which were highly enriched for functional pathways, such as “regulation of immune effector process” and “regulation of T cell activation” (**Figure 2D**). Direct comparison of CD4^+^ and CD8^+^ “TCR-activated” clusters revealed the common and different killing properties of cytotoxic CD4^+^ TCR-Ts and CD8^+^ TCR-Ts. Classical cytotoxic mechanisms including granule-dependent and FAS/FASLG signaling pathways were commonly adopted by CD4^+^ and CD8^+^ TCR-Ts. Effector genes such as *GZMA*, *GZMB*, *IFNG, TNF, IL-2* and corresponding pathways were significantly upregulated in CD4^+^ and CD8^+^ TCR-Ts, suggesting antigen induced activation of TCR signaling pathways (**Figures 3A, B**), similar as the previous report (43).

Specifically, our results revealed that compared with CD8^+^ TCR-Ts, cytotoxic CD4^+^ TCR-Ts highly expressed LT-α (**Figures 3A, D**), a TNF superfamily cytokine produced by lymphocytes, which mediates a variety of inflammatory, immunostimulatory and antiviral responses. Though LT-α was found to induce the apoptosis of a wide range of tumor cells (44), its role in antitumor responses with CD4^+^ T cells has not been recognized. According to the cellular communication analysis, LT-α from CD4^+^ TCR-Ts displayed significant interactions with its receptors, TNFRSF14, TNFRSF1A and TNFRSF1B on target cells (**Figure 3C**), strongly suggesting that LT-α functioned as effector molecules for cytotoxic CD4^+^ TCR-Ts. Among clusters (CTL1/2/3/4) of cytotoxic CD4^+^ TCR-Ts, expression levels of *LTA* had an increasing trend towards the later stage of CTL clusters (**Figure 4G**). It was also suggested that the expression of *LTA* positively correlated with prolonged survival of melanoma patients (**Figure 4H**), underlining the importance of *LTA* in antitumor immune responses.

The functional transition of CD4^+^ TCR-Ts to acquire cytotoxic activity seemed to be shaped by several transcription factors (**Figure 4F**), which was also reflected by previous studies (32–34). We observed significant upregulation of *GATA3*, *RUNX3*, and *TBX21*, *PRDM1* to a lesser extent, accompanied by the downregulation of *TCF7*, supporting the differentiation paths of CD4^+^ TCR-Ts towards mixed Th1/Th2 phenotypes with acquisition of cytotoxic functions, resembling the findings on chimeric antigen receptor-engineered T (CAR-T) cells (45, 46). This adds to the lineage plasticity of CD4^+^ T cells, as both Th1 or Th2 cells can be polarized to cytotoxic T cells in response to extrinsic factors (10, 47). Interestingly, the expression levels of *PRDM1* were elevated in CTL1/2/4 and then dropped in CTL3, suggesting that *PRDM1* might be associated with the initiation of CD4^+^ cytotoxic program, but not required for the sustained cytotoxic functions. This suggests that the generation of cytotoxic CD4 T cells may be a complex and ongoing dynamic process.

The incorporation of CD4^+^ TCR-Ts into tumor immunotherapy is expected to present several advantages. In addition to the direct cytotoxic effect described in this work and previous studies, CD4^+^ TCR-Ts produced large amount of IL-2 (**Figure 3D**), which could provide proliferative signals for CD8^+^ T cells *in vivo* (48). Various costimulatory receptors were highly expressed in activated CD4^+^ TCR-Ts (**Figure 4F**), which might promote strong antitumor CD8^+^ T cell responses through B cells or DCs (49, 50). Some studies suggested the long-term efficacies of CD4^+^ T cells were superior to CD8^+^ T cells, either using a trispecific T cell engager targeting HER2, CD3 and CD28 (26), or engineered as CAR-T cells (51, 52). A clinical study of adoptive cell therapy for leukemia found that CD4^+^ CAR-T dominated the late CAR-T cell population (more than 95%) which persisted for up to 10 years, and single-cell analysis showed that these long-lived CD4^+^ CAR-T cells exhibited cytotoxicity characteristics and sustained functional activation and proliferation (52). The underlying mechanisms are not clear yet, nevertheless, these data indicated that CD4^+^ T cells might hold potential for long-term tumor control. Future investigation on the persistence of CD4^+^ TCR-Ts *in vivo* is awaited, as well as means to promote the memory formation to achieve enduring antitumor effect. Besides, CD4^+^ T cells roughly accounted for 20% of human PBMC and are natural host for HIV-derived viral vectors, with higher transduction efficiency than CD8^+^ T cells, providing an additional resource for manufacturing cell products (20).

Some limitations for CD4^+^ TCR-Ts as the adoptive cell therapy exist. First, CD4^+^ T cells might have a slower onset of action to exert cytotoxic functions, as demonstrated in our study overexpressing an MHC class I-restricted TCR (**Figure S1C**) and in Cachot et al’s work using CD4^+^ CTLs targeting an MHC class II-restricted epitope (37). Second, identification of antigen-specific TCRs for engineering CD4^+^ T cells poses another challenge with low expression of MHC class II molecules (13, 14) and immune editing of MHC class II-restricted epitopes by tumors (42). As discussed above, CD4^+^ TCR-Ts targeting MHC class I-restricted tumor epitopes might serve as complementary therapeutics to CD8^+^ T cells.

Care needs to be taken when comparing our data with T cells in tumor microenvironment, which might be different from the *in vitro* analysis. scRNA sequencing of tumor infiltrating T cells suggested that tumor-specific T cells developed exhausted phenotypes with high expression of multiple inhibitory molecules (53, 54), while our results mostly reflected the cytotoxic states upon antigen stimulation for a short time. Future studies involving samples from murine models, human tumor organoid models or clinical trials are of great interest to uncover the functional states and regulatory mechanisms of cytotoxic CD4^+^ TCR-Ts *in vivo*.

Recognizing the important role of CD4^+^ T cells in mediating cancer immunity and understanding the biology of cytotoxic CD4^+^ T cells will lead to novel approaches to further enhance their direct antitumor activity in patients. Collectively, our study characterized the functional and transcriptomic profiles of MHC class I-restricted cytotoxic CD4^+^ TCR-Ts, and revealed the multifaceted killing mechanisms of this T cell subset, suggesting that they could serve as potent cancer immunotherapies.

## Materials and Methods

### Peptide

MART-1_27-35_ peptide LAGIGILTV (HLA-A:0201) was synthesized by GenScript (Nanjing, China) and the purity is higher than 99.0%. Peptide was stored at -20°C at a concentration of 10 mg/mL in 100% dimethyl sulfoxide (DMSO; Sigma-Aldrich, USA).

### Cell Lines

T2, 293T and Jurkat cell lines were purchased from the American Tissue Culture Collection (ATCC, USA). Melanoma cell line A375 was purchased from Beina Chuanglian Biotechnology (Beijing, China). A375 was infected by lentivirus expressing MART-1_27-35_ minigene and subcloned by limited dilution to generate MART-1_27-35_-overexpressing A375 (A375_MART-1_). Minigene construction followed the previously described method (55). T2 was cultured in IMDM (Gibco™, USA). 293T and A375_MART-1_ were maintained in DMEM (Gibco™, USA). Jurkat was cultured in RPMI (Gibco™, USA). Culture mediums for cell lines were supplemented with 10% heat-inactivated fetal bovine serum (FBS; Hy Clone™, USA), 100 units/mL penicillin, and 100 μg/mL streptomycin (Gibco™, USA). All cell cultures were maintained in a humidified incubator at 37°C with 5% CO_2_.

### Generation of MART-1_27-35_-Specific T Cells

Human peripheral blood mononuclear cells (PBMC) were obtained from HLA-A:0201 healthy donors in accordance with Institutional Review Board-approved protocols. CD14^+^ monocytes and CD8^+^ T cells were isolated from PBMCs by magnetic beads (Miltenyi Biotec, Germany). CD14^+^ cells were resuspended at 1×10^6^ cells/mL using X-VIVO15 (Lonza, Switzerland) containing 2% FBS (HyClone™, USA), 800 U/mL GM-CSF, 1000 U/mL IL-4 and cultured in 6-well plate, with medium replacement every two days. After 4 days, replace the medium with fresh X-VIVO15 containing 2% FBS, 20 ng/mL IL-6, 20 ng/mL IL-1β, 40 ng/mL TNF-α, 1 μg/mL PGE-2 (Sigma-Aldrich, USA), 800 U/mL GM-CSF and 1000 U/mL IL-4, and continued culturing for 2 days to allow DC maturation. Then DCs were loaded with MART-1_27-35_ at 10 μg/mL overnight, and were subsequently co-cultured with CD8^+^ T cells at DC:T ratio of 1:5 in the T009 medium ((BIOENGINE Sci-Tech, Shanghai, China) with 2% FBS and 30 ng/mL IL-21 for 48-72 h. CD8^+^ T cells were then transferred to a new plate with T cell growth medium (T009 containing 2% FBS, 10 ng/mL IL-2, 10 ng/mL IL-7 and 10 ng/mL IL-15), expanded for one week, followed by another round of DC stimulation and *in vitro* expansion. Cytokines were purchased from Peprotech, USA. Culture mediums for primary cells were supplemented with 100 units/mL penicillin, and 100 μg/mL streptomycin.

### IFN- γ Enzyme-Linked Immunospot (ELISPOT) Assay

IFN-γ ELISPOT assay was used to measure IFN-γ secretion from T cells. Briefly, T cells were rested without cytokines for 24 h. T2 cells were pulsed with MART-1_27-35_ peptide at a concentration of 10 μg/mL or DMSO overnight. 2×10^4^ T cells were seeded per well in a 96-well ELISPOT plate (Mabtech, Sweden), and co-cultured with 5×10^3^ T2 cells pulsed with MART-1_27-35_ peptide or DMSO for 20 h. All co-cultures of T cells with T2 cells were maintained in T009 with 2% FBS without adding exogenous cytokines. Detection of immunospots was performed according to the kit manual. Immunospots were imaged and read using an ELISPOT reader AID iSpot (AID-Autoimmun Diagnostika GmbH, Strassberg, Germany).

### Flow Cytometry

To sort MART-1_27-35_-specific T cells, CD8^+^ CTLs were labeled with FITC-conjugated anti-CD8-antibody (BD biosciences, USA) and APC-conjugated MART-1_27-35_-MHC tetramers. Tetramers were generated *via* UV light-induced cleavage of MHC-bound ligands from Flex-T™ monomer UVX (Biolegend, USA) as described previously (56). To sort exogenous TCR-positive cells, TCR-Ts were labeled with APC-conjugated anti-mouse TCRβ-antibody (Biolegend, USA). Staining of cell surface proteins were performed in flow buffer (phosphate buffered saline (PBS) +0.5% FBS) at 4°C in dark for 30 min. After staining, cells were washed and resuspended in flow buffer.

FACS sorting or flow cytometric analysis was performed using a FACS Arial II flow cytometer (Becton Dickinson, USA). Flow cytometry data was analyzed using FlowJo (FlowJo Enterprise, USA).

### ScRNA and V(D)J Sequencing

scRNA and V(D)J sequencing were performed as described previously (56, 57). Briefly, cell suspensions of sorted MART-1_27-35_-specific T cells were loaded onto a GemCode Single-Cell instrument (10x Genomics, Pleasanton, CA) to generate a single-cell emulsion. scRNA-seq libraries and TCR enriched libraries were prepared according to the instructions of 10x Genomics kit. Single-cell barcoded cDNA libraries and TCR libraries were then sequenced on BGI-seq 500 (MGI, Shenzhen).

### Lentivirus Vectors Packaging

Lentiviral vectors for TCR expression were generated as previously described (56). Basically, the genes encoding TCR variable regions of TCR from single-cell V(D)J sequencing were fused with murine TCRα and β2 constant regions, respectively. The TCRα and β chains were linked by a P2A self-cleaving peptide. The TCR expression cassette was codon optimized, synthesized (GenScript, Nanjing, China), and cloned into the pRRLSIN.cPPT.PGK-GFP.WPRE lentiviral vector (Addgene, USA). TCR-encoding lentivirus were collected from 293T cells transfected with the transfer plasmid and the packaging plasmids PsPAX2, pMD2.G (Addgene, USA). 48 h and 72 h after transfection, cell supernatants were collected and filtered through 0.45 μm syringe filters (Sartorius, Germany). Virus particles were pelleted by ultracentrifugation (Beckman Coulter, USA), resuspended in T009, aliquoted on ice, and stored at -80°C.

To determine the viral infection titer, jurkat cells were infected with the lentivirus at different volumes, and the expression of exogenous TCR-β after transduction was detected by flow cytometry. The infection titer is calculated using the formula: Infection titer (IU/mL) = cell number at infection time × positive rate/virus volume.

### Transduction of T Cells and Culture of TCR-Ts

To determine the appropriate MOI (Multiplicity of infection) to transduce primary T cells, primary CD4^+^ or CD8^+^ T cells were infected by lentirvus at different MOIs, then the expression of exogenous TCR-β were detected by flow cytometry. As in this study, we found T cells infected at MOI of 1-3 had highest expression of exogenous TCR-β and maintained high cell viability and proliferation rate.

CD4^+^ T cells and CD8^+^ T cells were isolated from PBMCs by magnetic beads (Miltenyi Biotec, Germany), activated by anti-CD3/CD28 microbeads (Miltenyi Biotec, Germany) in T009 containing 2% FBS and 10 ng/mL IL-2. After 48 h, cells were mixed with lentivirus in the presence of 6 μg/mL polybrene (Sigma-Aldrich, USA), and centrifuged at 800 g for 30 min at room temperature. 24 h after transduction, the medium was replaced with fresh T cell growth medium (T009 containing 2% FBS, 10 ng/mL IL-2, 10 ng/mL IL-7 and 10 ng/mL IL-15) and T cells were expanded for 4 days before measuring transduction efficiencies. For functional studies involving cytokine release, cytotoxicity, mouse studies or scRNA seq, T cells were expanded in culture for another 7-10 days.

### Intracellular Cytokine Staining

CD4^+^ or CD8^+^ TCR-Ts were rested without cytokines added for 24 h. Then 2×10^5^ CD4^+^ or CD8^+^ TCR-Ts were co-cultured with 5×10^4^ T2 cells pulsed with MART-1_27-35_ peptide or DMSO for 5 h, and Golgi blocker was added at the beginning of incubation (BD biosciences, USA). After stimulation, surface markers including CD4, CD8, or TCR were stained using FITC-conjugated anti-CD4-antibody, FITC-conjugated anti-CD8-antibody, PE-conjugated anti-mouse TCRβ-antibody, respectively. After washing with flow buffer, cells were fixed using fixation buffer (BD biosciences, USA), then permeabilized using 1x perm/wash buffer (BD biosciences, USA) according to manufacturer’s recommendations. PE-cy7-conjugated anti–IFN-γ–antibody, APC-conjugated anti-TNF-antibody were used to label intracellular cytokines. After 30 min incubation at 4°C, cells were washed with 1x perm/wash buffer twice and finally resuspended in flow buffer. Cells were acquired using a FACS Arial II flow cytometer. Antibodies were purchased from BD biosciences.

### CFSE Cytotoxicity Assay

T2 cells pulsed with MART-1_27-35_ or DMSO were stained with 5 μM CFSE (Invitrogen™, USA) for 10 min, then washed twice to remove excess CFSE. TCR-Ts and T2 were incubated for 6h at effector-to-target ratios of 10:1 or 2:1. After incubation, cells were pipetted out, and stained by PI (Invitrogen™, USA) just before flow cytometric analysis to label dead cells. Percentage of cytotoxicity was calculated as below:


$$\text{Cytotoxicity}\%\;=\;\left({\text{Percentage of CFSE}}^{+}{\text{PI}}^{+}\text{cells}\right)/\left({\text{Percentage of total CFSE}}^{+}\text{cells}\right)\;\times \;100\%$$

### LDH Cytotoxicity Assay

CD4^+^ or CD8^+^ TCR-Ts were incubated with A375 _MART-1_ for 24h at effector-to-target ratios of 20:1 or 1:1. Culture supernatants were transferred to a 96-well plate and LDH activity was quantified using the CytoTox96 Non-Radioactive Cytotoxicity Assay (Promega, USA) following manufacturer’s protocols. Percentage of cytotoxicity was calculated as below: Cytotoxicity% = (Experimental – Effector Spontaneous – Target Spontaneous)/(Target Maximum – Target Spontaneous) × 100%.

### RTCA

Impedance-based real-time cell analysis (RTCA; Agilent, USA) was used to monitor cell growth in a real-time manner as described (58). Briefly, 2×10^3^ A375_MART-1_ were seeded in each well of the 96-well E-plate (Agilent, USA). After 24 h, TCR-Ts were added to designated wells at effector-to-target ratio of 3:1. Growth of A375_MART-1_ with or without TCR-Ts was monitored by RTCA every 15 min for 47 h.

### Mouse Xenograft Models

The mouse experiments were performed at WuXi AppTec (Suzhou, China) according to the institutional protocols and the national laws and regulations. All animal studies were reviewed and approved by the Institutional Animal Care and Use Committee (IACUC) of WuXi AppTec (Approval# SZ20210421-Mice-A).

Adult female NOG mice (NOD.Cg-Prkdc^scid^IL2rg^tmlSug^/JicCrl) were inoculated subcutaneously with 3×10^6^ A375 _MART-1_ cells. Once the tumors reached the volume of 50-100 mm^3^ (~1 week after injection), mice were treated with peritumoral injection of 3×10^7^ TCR-T cells, or mock T cells (transduced with lentivirus carrying GFP gene) from the same donor, or PBS as control. After 1 week, the second dose was injected. Mice weight and tumor size was measured every two days, and tumor volume was calculated by length×width^2^/2. Mice were sacrificed when tumor size reached 2000 mm³ or signs of distress was observed as determined in the animal experimentation protocol.

### TCR-Ts Stimulation With T2 Cells for scRNA Sequencing

CD4^+^ or CD8^+^ TCR-Ts were prepared as described above, rested without exogenous cytokines added for 24 h before coculture with T2 cells. Then CD4^+^ or CD8^+^ TCR-Ts were co-cultured with T2 cells pulsed with MART-1_27-35_ peptide or T2 cells pulsed with DMSO for 3h or 6h in a round bottom 96-well plate. The culture was set at 1×10^6^ cells/mL, effector-to-target ratio of 10:1 in 200 μL/well. After stimulation, the co-culture was gently mixed, pipetted out, washed and subjected to scRNA-seq.

### Cytokine Release Measurement

CD4^+^ TCR-Ts or CD8^+^ TCR-Ts were incubated with MART-1_27-35_-pulsed or DMSO-loaded T2 for 6/24/48 h at an effector-to-target ratio of 1:1. After incubation, supernatants were collected and subjected to cytokine multiplex analysis using the human Luminex discovery assay (R&D systems, USA) in accordance with the manufacturer’s instructions. The cytokine levels were determined using the Luminex 200 System (Luminex Corporation, USA).

### Single-Cell RNA-Seq Data Pre-Processing

Raw files were processed using Cell Ranger (version 3.0.2, 10 x Genomics, USA) with default arguments except for setting “except-cells” to 6000. Reads were aligned and quantified against the GRCh38 human reference genome provided by Cell Ranger.

### Quality Control

For each sample, the UMI count matrix was loaded into R (version 3.6.3) and processed by using the Seurat package (version 3.2.1) (59). Tukey’s method (60) was applied to exclude cells with outliers of gene number. The data of eight samples were log normalized separately with the “NormalizeData” function and then integrated by using the “merge” function with default parameters. The integrated data was pre-clustered into 14 clusters by using 4000 variable genes, the first 40 principal components, and resolution 0.2. The cell types were determined according to the expression of marker genes. To filter broken or dying T cells, T cell clusters with fewer detected genes or higher percentages of mitochondrial genes were excluded.

### Clustering and Cell Annotation

After filtering, 4000 variable genes were reselected with the “FindVariableFeatures” function and further reduced into 50 principal components by using the “RunPCA” function. Cells were finally clustered into 11 clusters at 0.2 resolution based on the first 40 principal components. Cell types of clusters were assigned using the expression of known marker genes. Notably, one cluster was defined as the apoptotic cell (APT) since there is no marker gene expressed but with a high percentage of mitochondrial genes and few detected genes.

### TCR-T Cell Identification

To identify the TCR-T cells, we constructed a reference genome based on the lentiviral and TCR clonotype4 sequence. Then, raw reads were mapped and counted against this genome using Cell Ranger “count” command with default parameters. According to the UMI matrix obtained in the last step, any cell expressing any gene in the genome was defined as a TCR-T cell.

### Correlation Analysis

The average expressions of all detected genes in each sample were calculated by using the “Average Expression” function. Spearman’s rank correlation coefficients among samples were then calculated and used for hierarchical clustering with complete linkage as the clustering method and Euclidean distance as the measurement method.

### Differential Expression Analysis

Differential expression analysis was performed based on Wilcoxon rank-sum test. A 1.5-fold change in expression levels and a *P* value less than 0.05 were used as cutoffs to define differentially expressed genes.

### Gene Set Enrichment Analysis

Three gene set analyses were performed: Gene Ontology (GO) analysis, Gene Set Enrichment Analysis (GSEA), and analysis of gene set activity. GO analysis and GSEA were performed using the clusterProfiler package (61). A *P* value less than 0.05 was considered to be statistically significant. The R package AUCell (62) was used to calculate activity scores of manually curated get sets (provided in **Table S** in the supplementary material) (43) in T cells. The results were visualized with ggplot2 (63) based on the t-SNE coordinates extracted from the Seurat object.

### Trajectory Analysis

Trajectory analysis for CD4^+^ T cells and CD8^+^ T cells was carried out independently by using the monocle R package (version 2.14.0) (64). The raw UMI counts extracted from the Seurat object were used as expression inputs. The structure of inferred trajectory was visualized in 2-dimensional space using the monocle built-in method DDRTree.

### Cell-Cell Communication Analysis

Cell-cell communication analysis was performed sample by sample using CellPhoneDB (version 2.0) (65). To ensure accuracy, we filtered out cells in CD4 groups but annotated as CD8^+^ T cells, and cells in CD8 groups but annotated as CD4^+^ T cells. To obtain ligand-receptor pairs specifically indicating the regulations of functional T cells for target cells, we used the following criteria: Ligands and receptors expressed in at least 10% of cells in a given cell group were included, or the interactions were considered non-existent; only significant ligand-receptor pairs (*P* < 0.05) with clear annotation (ligand expressed in T cells, receptor expressed in target cells) were further analyzed; only consider ligand-receptor pairs existed between functional T cells and target cells and excluded those existed between background T cells and target cells.

### Single-Cell Regulatory Network Analysis

Single-cell regulatory network inference was performed based on scRNA-seq data using SCENIC (version 1.1.3) (62) with default parameters. Subsequently, the activity scores of each regulon were transformed to binary (activated/non-activated) using the “runSCENIC_4_aucell_binarize” function. The following criteria were applied to screening regulons related to T cell functions: regulons with high confidence were included in further analysis; manually curated pathways (**Table S**) were selected from GO analysis results. Regulons targeting genes in these pathways were considered as T cell function-related regulons; regulons activated in less than 30% of cells of given cell groups were excluded; the average activity scores of the filtered regulons in each cell group were calculated and visualized by using the ComplexHeatmap R package.

### *LTA* Downstream Target Gene Analysis

The potential downstream target genes of *LTA* downloaded from CytoSig (https://cytosig.ccr.cancer.gov/) were up-regulated genes under the *LTA* treatment with p < 0.05 in the “Whole BloodHealthy Adult” condition (GSE103500). The “FindMarkers” function of Seurat was used to identify the specifically up-regulated genes in CD4^+^ MART-1 T2 (CD4^+^ MART-1 T2 versus CD4^+^ DMSO T2 and CD8^+^ MART-1 T2 versus CD8^+^ DMSO T2). Finally, 10 overlapping genes between specifically up-regulated genes in T2 of CD4^+^ MART-1 and CytoSig were plotted for expression analysis.

### Statistical Analysis

Unless stated elsewhere, all experiments were performed in triplicates. The Student’s paired t-test was used for statistical analysis. Analyses were performed with Graph Pad (version 5.03; Graph Pad, Graph Pad Software Inc., CA, USA).

### Data Access

All raw and processed sequencing data generated in this study have been submitted to the CNGB Sequence Archive (CNSA; https://db.cngb.org/cnsa/) (doi:10.1093/database/baaa055) of the China National GeneBank DataBase (CNGBdb) (doi:10.16288/j.yczz.20-080) under accession number CNP0002524.

## Data Availability Statement

The data that support the findings of this study have been deposited into the CNGB Sequence Archive of CNGBdb with accession number CNP0002524 (https://db.cngb.org/search/?q=CNP0002524).

## Ethics Statement

The studies involving human samples were reviewed and approved by ethical clearance from the institutional review board of BGI. The participants provided their written informed consent to participate in this study. All animal studies were reviewed and approved by the Institutional Animal Care and Use Committee (IACUC) of WuXi AppTe. Number of IACUC Approval(SZ20210421-Mice-A).

## Author Contributions

LZ, H-XS, and QX designed the project and wrote the manuscript, and revised the manuscript with FM. YLL conducted the TCR-T experiments and assisted with thebioinformatic analysis and manuscript preparation. SL and JL performed the bioinformatic analysis. QX, FW and ZL performed experiments related to single-cell sequencing. LL, YTL and YJL helped with the project. LZ and FM supervised the project. All authors contributed to the article and approved the submitted version.

## Funding

This project is supported by the Science, Technology andInnovation Commission of Shenzhen Municipality (JSGG20180508152912700), Open Fund of Shenzhen Bay Laboratory (SZBL2020090501005) and Key Project of Shenzhen Bay Laboratory (S201101004).

## Conflict of Interest

The authors declare that the research was conducted in the absence of any commercial or financial relationships that could be construed as a potential conflict of interest.

## Publisher’s Note

All claims expressed in this article are solely those of the authors and do not necessarily represent those of their affiliated organizations, or those of the publisher, the editors and the reviewers. Any product that may be evaluated in this article, or claim that may be made by its manufacturer, is not guaranteed or endorsed by the publisher.

## Supplementary Material

Supplementary Table 1 Top4 MART-1_27-35_-specific TCR clonotypes

Supplementary Table 2 Sample information for scRNA-seq

Supplementary Figure 1 Construction of MART-1_27-35_-specific TCR-Ts and validation of their anti-tumor effects *in vitro* and *in vivo*. **(A)** Schematic diagram of the recombinant lentiviral vectors for TCR expression in this study. The GFP construct served as a negative control. **(B)** Flow cytometric analysis shows expression of exogenous TCR on 8 different TCR-T samples. **(C)** Real-time cell analysis shows the cell-index growth curve of A375_MART-1_ with different treatments. **(D) (Left)** The schematic diagram of mouse experimental design. NOG mice receive subcutaneous implantation of 3 × 10^6^ A375_MART-1_ cells on day -7. After 7 days or tumor reaches 50-100 mm^3^, mice were divided into 5 groups for different treatments on day 0 and day 7. **(Right)** Tumor growth inhibition rates by CD4^+^ or CD8^+^ TCR-Ts are calculated by tumor growth inhibition (TGI) (%)=(Cx-Tx)/Cx×100% (Cx is The average volume of the control group, Tx is the average volume of the experimental group) were plotted accordingly.

Supplementary Figure 2 Functional characterization of “TCR-activated” cytotoxic clusters. **(A)** Heatmap shows distinct transcriptomic profiles among CD4^+^ and CD8^+^ clusters (top5 DEGs displayed for each cluster). **(B)** Volcano plots display the differential expression genes of CD4^+^_C3/C4 or CD8^+^_C3/C4 compared with their remaining clusters. **(C-E)** Activity scores of representative gene sets for activation/exhaustion **(C)**, co-stimulation **(D)** or cytotoxicity **(E)** illustrated in t-SNE plots and compared in histograms for CD4^+^ and CD8^+^ clusters. Error bar denote SEM. *: P < 0.05, **: P < 0.01, ***: P < 0.001. (Wilcoxon test).

Supplementary Figure 3 CD4^+^ and CD8^+^ TCR-Ts activate apoptosis pathways in T2 target cells. **(A)** Venn diagram of up-regulated genes in CD4^+^_C3/C4 and CD8^+^_C3/C4 compared with their remaining clusters. **(B)** GSEA of different apoptosis pathways in the target cells incubated with CD4^+^ TCR-Ts or CD8^+^ TCR-Ts. **(C, D)** GSEA of apoptosis pathways in target cells of treatment groups CD4^+^ TCR-Ts **(C)** or CD8^+^ TCR-Ts **(D)**. **(E)** The heatmap showing activities of transcription regulons in CD4^+^ and CD8^+^ clusters. **(F)** Cytokine secretion from TCR-Ts stimulated with MART-1_27-35_ or DMSO-pulsed T2 cells measured by Luminex. Groups were compared with a two-sided, unpaired t-test. *: P < 0.05, **: P < 0.01, ***: P < 0.001. GSEA, gene set enrichment analyses.

Supplementary Figure 4 Functional characterization of CTLs. **(A-D)** GO analysis using top 30 DEGs depicting pathways significantly enriched in the CTL1 **(A)**, CTL2 **(B)**, CTL3 **(C)** and CTL4 **(D)** compared with other clusters. Top5 GO-BP terms were displayed for each cluster. The color key from red to blue indicates P values from low to high. Count: The number of genes enriched to this GO entry from the input gene for enrichment analysis. GO, gene ontology. BP, biological process.

## Abbreviations

DMSO, Dimethyl sulfoxide; MHC, Major histocompatibility complex; TCR-T, T cell receptor engineered T cell; CDR, Complementarity determining region; CTL, Cytotoxic T lymphocytes; DC, Dendritic cell; UMAP, Uniform approximation and projection; DEG, Differential expressed gene; TAAs, Tumor associated antigens; MART-1, Melanoma antigen recognized by T-cells 1; HLA-A2, Human leukocyte antigen-A2; IFN-γ, Interferon γ; TNF-α, Tumor necrosis factor-α; LT-α, Lymphotoxin-α; GFP, Green Fluorescent Protein; MS4A1, Recombinant Membrane Spanning 4 Domains Subfamily A, Member 1; TAP1, Antigen Peptide Transporter 1; CD40LG, Clusters of differentiation 40 ligand; LTA, Lymphotoxin A; IFNG, Interferon gamma gene; TNF, Tumor necrosis factor; PRDM1, Positive regulatory domain I-binding factor 1; FASLG, Fas ligand; SLAMF7, Signaling Lymphocytic Activation Molecule Family, Member 7; TCGA, The Cancer Genome Atlas; CAR-T, Chimeric Antigen Receptor T Cell; GSEA, Gene set enrichment analysis; JAK-STAT, Janus kinase-signal transducers and activators of transcription; GITR, Glucocorticoid-induced tumor necrosis factor receptor; TNFRSF14, Tumor necrosis factor receptor superfamily, member 14; IRF4, Interferon Regulatory Factor 4; IRF8, Interferon Regulatory Factor 8; TNFRSF1B, Tumor necrosis factor receptor superfamily member 1B; TRAIL, TNF-related apoptosis-inducing ligand; GO, Gene Ontology; BP, Biological process; RUNX3, Runt-related transcription factor 3; LAMP1, Lysosome-associated membrane protein 1; NKG7, Natural killer cell granule protein 7; SKCM, Skin cutaneous melanoma; PBMC, Peripheral blood mononuclear cells; GM-CSF, Granulocyte-macrophage colony stimulating factor; FBS, Fetal bovine serum; ELISPOT, Enzyme-linked Immunospot Assay; PEI, Polyetherimide; UMI, Unique molecular identifiers; MOI, Multiplicity of infection.
