# Supplementary figures and images for: Single-Cell Transcriptomics Reveals Killing Mechanisms of Antitumor Cytotoxic CD4+ TCR-T Cells
Source: Front Immunol. 2022 Jul 19;13:939940. doi: 10.3389/fimmu.2022.939940 (PMC9343810; doi:10.3389/fimmu.2022.939940)

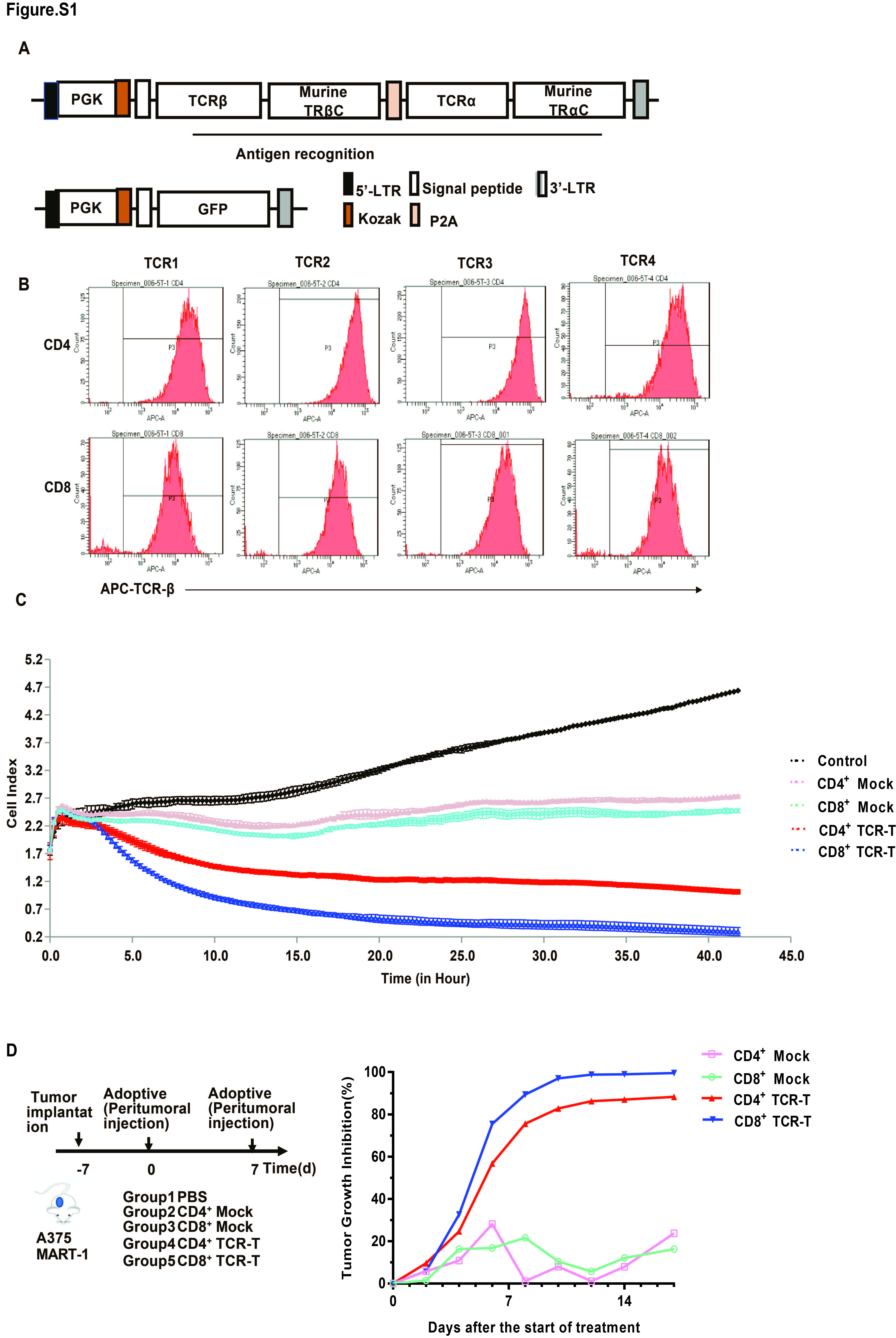

Supplement: Supplementary Figure 1 — Construction of MART-127-35-specific TCR-Ts and validation of their anti-tumor effects in vitro and in vivo. (A) Schematic diagram of the recombinant lentiviral vectors for TCR expression in this study. The GFP construct served as a negative control. (B) Flow cytometric analysis shows expression of exogenous TCR on 8 different TCR-T samples. (C) Real-time cell analysis shows the cell-index growth curve of A375MART-1 with different treatments. (D) (Left) The schematic diagram of mouse experimental design. NOG mice receive subcutaneous implantation of 3 × 10^6^ A375MART-1 cells on day -7. After 7 days or tumor reaches 50-100 mm3, mice were divided into 5 groups for different treatments on day 0 and day 7. (Right) Tumor growth inhibition rates by CD4+ or CD8+ TCR-Ts are calculated by tumor growth inhibition (TGI) (%)=(Cx-Tx)/Cx×100% (Cx is The average volume of the control group, Tx is the average volume of the experimental group) were plotted accordingly. [file Image_1.jpeg]

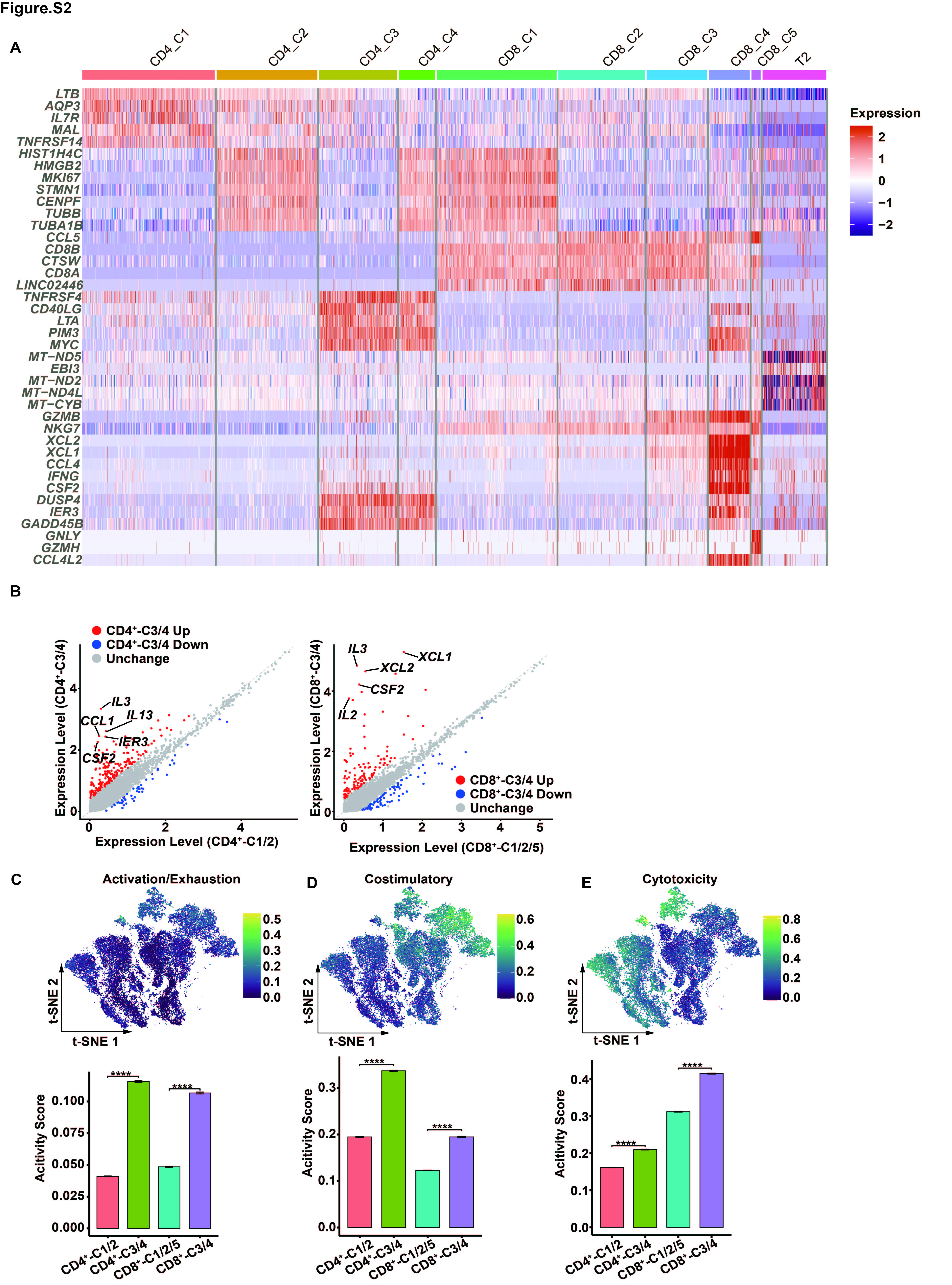

Supplement: Supplementary Figure 2 — Functional characterization of “TCR-activated” cytotoxic clusters. (A) Heatmap shows distinct transcriptomic profiles among CD4+ and CD8+ clusters (top5 DEGs displayed for each cluster). (B) Volcano plots display the differential expression genes of CD4+_C3/C4 or CD8+_C3/C4 compared with their remaining clusters. (C-E) Activity scores of representative gene sets for activation/exhaustion (C), co-stimulation (D) or cytotoxicity (E) illustrated in t-SNE plots and compared in histograms for CD4+ and CD8+ clusters. Error bar denote SEM. *: P < 0.05, **: P < 0.01, ***: P < 0.001. (Wilcoxon test). [file Image_2.jpeg]

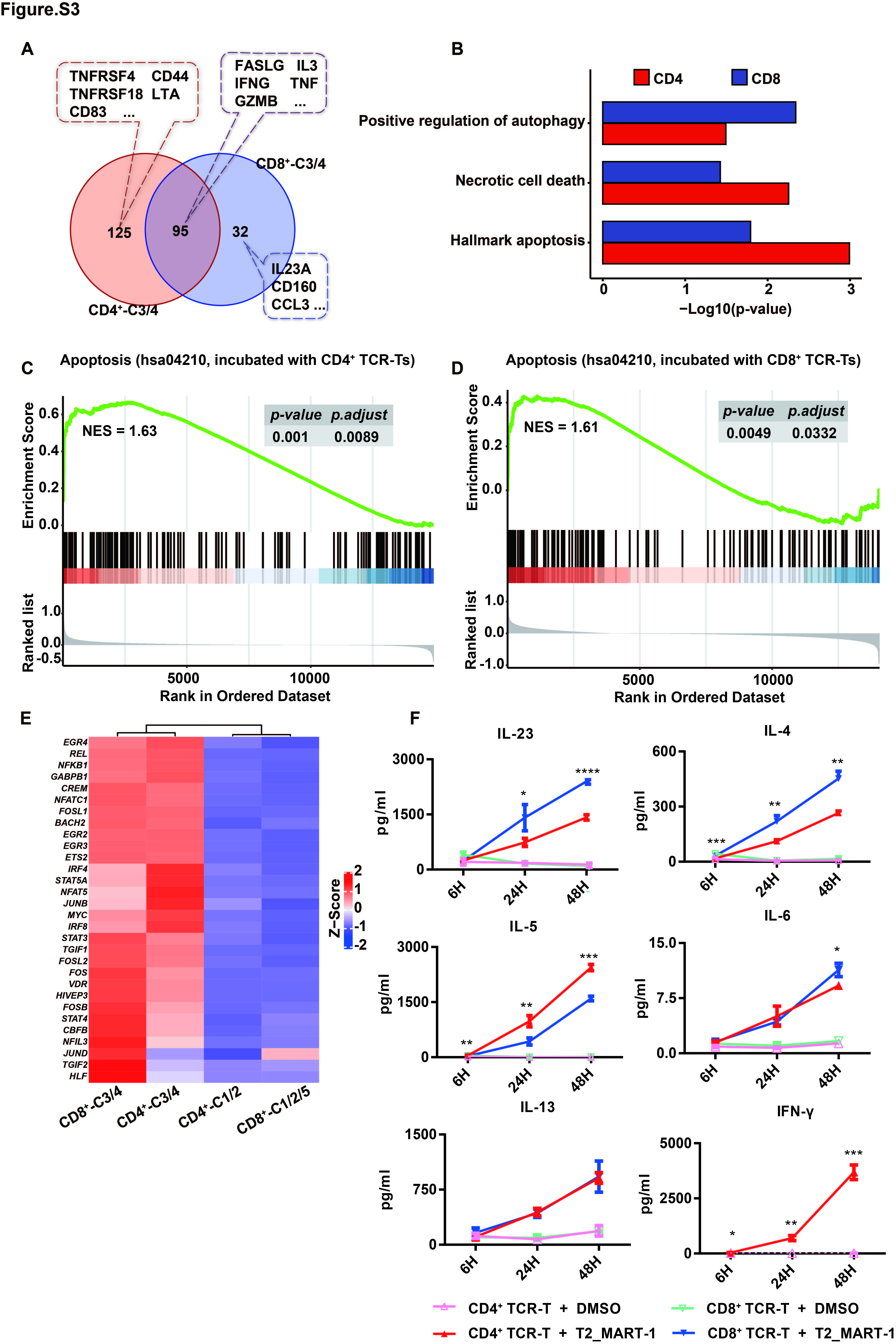

Supplement: Supplementary Figure 3 — CD4+ and CD8+ TCR-Ts activate apoptosis pathways in T2 target cells. (A) Venn diagram of up-regulated genes in CD4+_C3/C4 and CD8+_C3/C4 compared with their remaining clusters. (B) GSEA of different apoptosis pathways in the target cells incubated with CD4+ TCR-Ts or CD8+ TCR-Ts. (C, D) GSEA of apoptosis pathways in target cells of treatment groups CD4+ TCR-Ts (C) or CD8+ TCR-Ts (D). (E) The heatmap showing activities of transcription regulons in CD4+ and CD8+ clusters. (F) Cytokine secretion from TCR-Ts stimulated with MART-127-35 or DMSO-pulsed T2 cells measured by Luminex. Groups were compared with a two-sided, unpaired t-test. *: P < 0.05, **: P < 0.01, ***: P < 0.001. GSEA, gene set enrichment analyses. [file Image_3.jpeg]

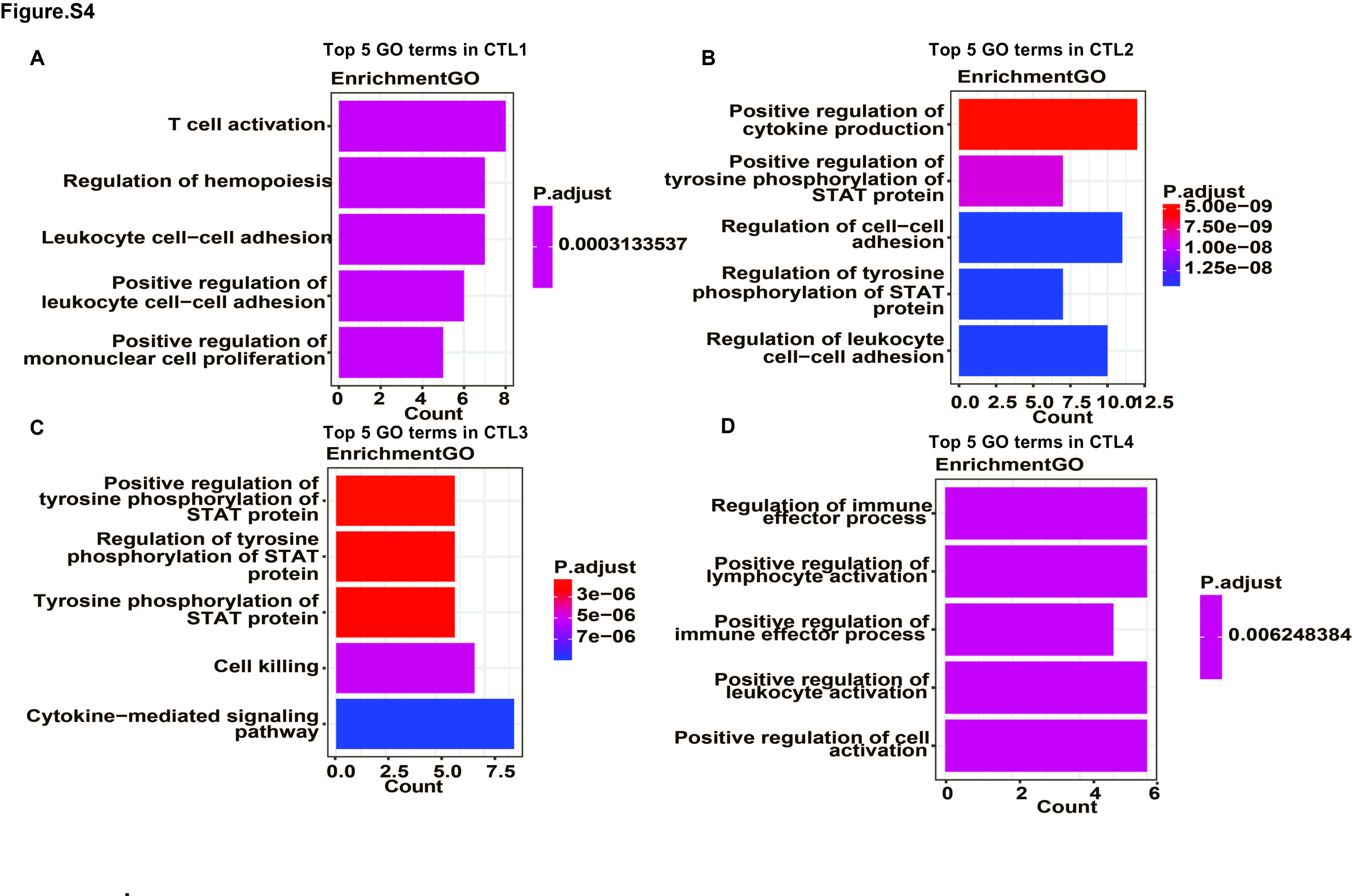

Supplement: Supplementary Figure 4 — Functional characterization of CTLs. (A-D) GO analysis using top 30 DEGs depicting pathways significantly enriched in the CTL1 (A), CTL2 (B), CTL3 (C) and CTL4 (D) compared with other clusters. Top5 GO-BP terms were displayed for each cluster. The color key from red to blue indicates P values from low to high. Count: The number of genes enriched to this GO entry from the input gene for enrichment analysis. GO, gene ontology. BP, biological process. [file Image_4.jpeg]
